# Transcriptome Analysis Reveals Effect of Dietary Probiotics on Immune Response Mechanism in Southern Catfish (*Silurus meridionalis*) in Response to *Plesiomonas shigelloides*

**DOI:** 10.3390/ani13030449

**Published:** 2023-01-28

**Authors:** Rongrong Wang, Jiaming Qian, Da Ji, Xingyu Liu, Ranran Dong

**Affiliations:** College of Animal Science, Guizhou University, Guiyang 550000, China

**Keywords:** probiotic complex, spleen, high-throughput sequencing, immune-related pathways

## Abstract

**Simple Summary:**

We probed the effect of a probiotic complex on the immune response caused by *Plesiomonas shigelloides* infection in the southern catfish. Our study revealed the key genes for this inflammatory response, and the probiotic complex effectively inhibited the expression of key inflammatory factors caused by *P. shigelloides*, providing basic data for future artificial breeding of the southern catfish and the prevention and control of bacterial diseases caused by *P. shigelloides*.

**Abstract:**

To explore whether a probiotic complex composed of *Lactobacillus rhamnosus*, *Lactobacillus plantarum*, and *Lactobacillus casei* can prevent or inhibit the inflammatory response caused by the invasion of *Plesiomonas shigelloides* in the southern catfish, we screened differentially expressed genes and enriched inflammation-related pathways among a control and three experimental groups and conducted analysis by transcriptome sequencing after a 21-day breeding experiment. Compared with those in the PS (*Plesiomonas shigelloides*) group, southern catfish in the L-PS (*Lactobacillus-Plesiomonas shigelloides*) group had no obvious haemorrhages or ulcerations. The results also showed that inflammation-related genes, such as *mmp9*, *cxcr4*, *nfkbia*, *socs3*, *il-8*, *pigr*, *tlr5*, and *tnfr1*, were significantly upregulated in the PS group compared with those in the L-PS groups. In addition, we verified six DEGs (*mmp9*, *cxcr4*, *nfkbia*, *socs3*, *rbp2*, and *calr*) and three proteins (CXCR4, NFKBIA, and CALR) by qRT-PCR and ELISA, respectively. Our results were consistent with the transcriptome data. Moreover, significantly downregulated genes (*p* < 0.05) were enriched in inflammation-related GO terms (lymphocyte chemotaxis and positive regulation of inflammatory response) and immune-related pathways (intestinal immune network for IgA production and IL-17 signalling pathway) in the L-PS vs. the PS group. Our results indicate that the infection of *P. shigelloides* can produce an inflammatory response, and probiotics could inhibit the inflammatory response caused by *P. shigelloides* to some extent.

## 1. Introduction

The goal of intensive aquaculture is to create the most suitable environment for the growth and development of aquatic organisms at high densities in small facilities by using advanced feeding management methods, achieving a high yield and efficiently breeding the organisms in a relatively short period of time by accelerating their growth rate to increase their production. However, the development of the aquaculture industry is restricted by animal diseases that are caused by the aquatic environment in high-density aquacultural facilities, which are more conducive to the reproduction, growth, and spread of bacteria. Therefore, bacterial diseases have seriously affected the economical profitability of the aquaculture industry [1].

The southern catfish (*Silurus meridionalis*) (Siluriformes, Siluridae) is an economically important species with a large size, high nutritional value [2], rapid growth [3,4,5], disease resistance [6], wide environmental adaptability [7], and euryphagous [8,9], and is widely cultured in China. Southern catfish can survive in sewage; however, despite being more disease-resistant than other fish, it is still unable to avoid the invasion of some bacterial diseases. At present, a set of pathogens have been isolated in southern catfish, such as *Vibrio vermiculo* [10], *Proteus commoner* [11], *Aeromonas guinea* [12], *Aeromonas hydrophila* [13], *Edwardsiella ctalurid* [14], *Aeromonas viridans* [15], *Edwardsia* [16], *Columnar flex* [17], *Pseudomonas fluorescens* [17], *Pseudomonas albicans* [17], *Cronobacter sakazakii* [6], and *Aeromonas mild* [18]. *Plesimonas shigelloides*, a man–fish zoonotic pathogenic that causes great losses in the aquaculture of many freshwater fish, was isolated from southern catfish in a pre-experiment. There are no studies on *P. shigelloides* in southern catfish thus far; therefore, a study into its pathogenic mechanisms is necessary.

To date, a large number of antibiotic drugs have been used to fight bacterial diseases in aquaculture, and the drug residues are causing harm to human health. Thus, it is urgent to find non-toxic alternatives that yield no side effects. The way probiotics antagonise pathogenic bacteria is mainly reflected in four aspects: competing with pathogenic bacteria for adhesion sites, copolymerising with pathogenic bacteria, producing antibacterial substances, and enhancing the body’s immunity. For example, *Bifidobacterium breve* has been shown to increase the production of anti-influenza IgG, inhibiting influenza virus infections [19]. Some clinical studies have shown that fermented milk containing *Lactobacillus casei* DN114001 can reduce the severity, duration, and incidence of acute diarrhoea in children [20,21,22]. A European trial showed that giving oral lactobacillus GG rehydration solution to children with acute diarrhoea resulted in a shorter duration of illness [23]. Therefore, we developed our study based on this idea.

*L. rhamnosus* is a probiotic with high acid and bile resistance, as well as strong adhesion, meaning it can effectively colonise human and animal intestines [24]. Studies have shown that intervention with *L. rhamnosus* can significantly (*p* < 0.05) reduce the proliferation of harmful bacteria, such as *Shigella*, in the intestines of mice with ulcerative colitis, as well as inhibit colonic inflammation [25]. *L. casei* and *L. plantarum* also exhibit strong probiotic properties, show strong resistance to acids and bile, and have strong antagonistic activity against *Salmonella paratyphi* in the gastrointestinal tract [26]. In addition, adding appropriate probiotics to fish feed can effectively enhance their immune system function and increase their resistance to various pathogenic infections [27,28]. For example, *Oncorhynchus mykiss* showed high resistance to pathogenic bacterial infection after feeding with different doses of *L. rhamnosus* for 51 days [29]. Considering that the three probiotics above can effectively colonise and survive in the intestinal tract, these three were selected to constitute our probiotic complex. In our study, we screened immune-related differentially expressed genes (DEGs) by transcriptome sequencing to explore whether probiotics can prevent or inhibit bacterial diseases caused by *P. shigelloides*. We verified six genes and three proteins by qRT-PCR and ELISA, respectively. Additionally, we also deeply analysed the inflammation-related pathways enriched by DEGs.

## 2. Materials and Methods

### 2.1. Experimental Bacterial Strain

We previously isolated *P. shigelloides* and *Lactobacillus plantarum* from diseased southern catfish and healthy carp, and purchased *L. casei* (BNCC134415) and *Lactobacillus rhamnosus* (BNCC134266) strains from the BeNa Culture Collection. For subsequent experiments, we cultured *P. shigelloides* in a tryptic soy broth (TSB) medium for 7 h (exponential stage) at 37 °C. We also cultured *L. plantarum*, *L. casei*, and *L. rhamnosus* in an MRS liquid medium for 24 h at 37 °C without oxygen. After collection and washing three times with sterilised phosphate-buffered saline (PBS, pH 7.4), we resuspended the bacteria in sterile saline water to obtain a concentration of 1.0 × 10^9^ CFU/mL.

### 2.2. Animal and Experimental Design

We obtained healthy adult southern catfish, which were approximately 20 centimetres long, from a fish farm located in Hechuan, Chongqing, China. We kept the fish in freshwater tanks at 24–27 °C in a breeding room before acclimatising them to laboratory conditions for 8 weeks prior to the experiments.

First, we divided the experimental fish into four groups (each with 20 individuals): (1) blank control group (BC), (2) *Lactobacillus* group (L), (3) *Lactobacillus + P. shigelloides* group (L-PS), and (4) *P. shigelloides* group (PS). Feeding probiotic complex: L group and L-PS group were administered a mix of *Lactobacillus* (1.0 × 10^9^ CFU/mL, 0.2 mL) by intragastric administration for 21 d, whereas the BC group and PS group were gavaged with normal saline (0.2 mL) to provide the same management stress. *P. shigelloides* infection test: After 21 d, the L-PS group and the PS group were injected intraperitoneally with *P. shigelloides* (1.0 × 10^9^ CFU/mL, 0.2 mL), whereas the BC group and the L group were replaced by normal saline (0.2 mL) to provide the same management stress [30,31,32,33,34]. We collected samples 72 h after injecting *P. shigelloides*. We obtained spleens from each group (3 samples per group), which we immediately snap-froze in liquid nitrogen and stored at −80 °C for subsequent testing.

### 2.3. RNA Extraction, Library Construction, and Sequencing

We extracted the total RNA of the spleens using a mirVana™ miRNA ISOlation Kit (Ambion-1561) (Thermo Fisher Scientific, Waltham, MA, USA) following the manufacturer’s instructions. We detected the purity (OD260/280), concentration, and absorption peak of the RNA by a Nano Drop 2000 (Thermo Fisher Scientific, Waltham, MA, USA), and then examined the quality of the RNA by 1% agarose gel electrophoresis.

We constructed the libraries using the TruSeq Stranded mRNA LT Sample Prep Kit (Illumia, San Diego, CA, USA) according to the manufacturer’s instructions. Then, sequencing was completed using the Illumina HiSeq X Ten platform by OE Biotech Co., Ltd. (Shanghai, China), which generated 150 bp paired-end reads.

### 2.4. Data Processing and Assembly

We processed the raw data (raw reads) using Trimmomatic [35] (parameters: LEADING: 3 TRAILING: 3 SLIDINGWINDOW: 4: 15 MINLEN: 50) to remove poly-N and low-quality reads, which provided clean reads. We mapped clean reads to the southern catfish reference genomes by hisat2 [36] (version: 2.2.1) (parameters: -x –ss –exon). We assembled and merged the transcripts by StringTie [37] (version: 1.3.0) (parameters: --merge -G).

### 2.5. Functional Annotation

We functionally annotated the transcripts by alignment of the transcripts with the NCBI nonredundant (NR), SwissProt, and clusters of orthologous groups for the eukaryotic complete genome (KOG) databases using Diamond. The threshold takes E-value < 1e-5 to find orthologs. We used the proteins with the highest hits to the transcripts to assign functional annotations.

### 2.6. Analysis of Differentially Expressed Genes (DEGs), Cluster Analysis, GO and KEGG Enrichment

We calculated the read counts and FPKM using featureCounts [38] (version: 2.0.0) (parameters: -p -a -t -g) and eXpress [39] (version: 1.5.1) (parameters: --rf-stranded). We identified DEGs using the R packages DESeq2 [40] (version: 1.38.2) (parameters: qvalue < 0.05, |log2FoldChange| > 1) and edgeR [41]. We performed GO enrichment and KEGG pathway enrichment analysis of the DEGs using R package clusterProfiler [42] (version: 4.6.0) based on the hypergeometric distribution. We used R packages ggplot2 [43] (version: 3.4.0) to draw the column and bubble diagrams of the significant enrichment terms.

### 2.7. Validation of DEGs by q-PCR

To test the RNA-Seq results, we selected six DEGs (*mmp9*, *socs3*, *nfkbia*, *cxcr4*, *rbp2*, and *calr*) for qRT-PCR analysis. The specific primers used are listed in Table 1, and we used β-actin and 18S rRNA as internal controls. We incubated reactions at 94 °C for 30 s, followed by 45 cycles of 94 °C for 5 s and 60 °C for 30 s. We performed melting curve analysis to verify the specific generation of the expected PCR product after the PCR cycles ended. We normalised the expression levels of mRNAs to β-actin, and we calculated 18S rRNA using the 2^−ΔΔCt^ method [44].

### 2.8. Validation of DEGs by ELISA

We analysed three proteins (CXCR4, CALR, and NFKBIA) by enzyme-linked immunosorbent assay (ELISA) following the manufacturer’s instructions (Enzyme-Free Biologics) to further verify the reliability of the transcriptome data. We drew the standard curve according to the concentration and absorbance values of the standard to obtain the corresponding calculation formula. Then, we entered the absorbance value of each sample into the formula to calculate the secretion of each protein.

## 3. Results

### 3.1. Clinical Symptoms

**Figure 1:**
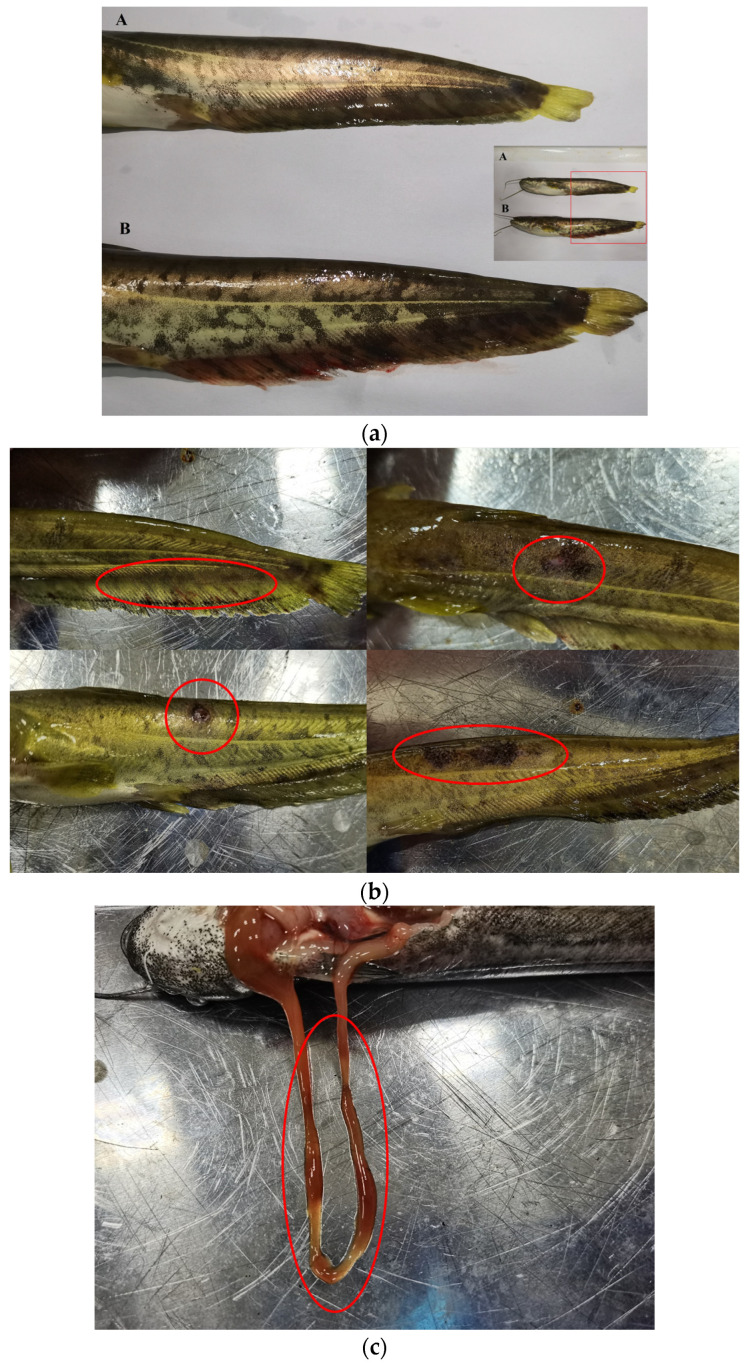
Body surface symptoms of southern catfish infected with *Plesiomonas shigelloides* for 72 h. (**a**) Bleeding of anal fin of southern catfish infected with *Plesiomonas shigelloides* in PS vs. L-PS group. A: Southern catfish in L-PS group; B: southern catfish in PS groups. (**b**) Body surface symptoms of southern catfish. (**c**) Intestinal redness and swelling of southern catfish.

In our experiment, we observed body surface changes in the PS and L-PS groups 72 h after injection of *P. shigelloides* (Figure 1a). The most obvious symptoms in the PS group were bleeding of the caudal fins and anal fin, and the blackening, moulting, and ulceration of the skin surface; however, we did not find lesions in other parts (Figure 1b). After dissection, we found one case of intestinal redness and swelling; however, no lesions were found in the other individuals (Figure 1c). In the L-PS group, except for two individuals with hyperaemia of the anal fin base and one with redness and swelling at the injection site, no lesions were found in other individuals. The observations showed that the BC and L groups showed no body surface symptoms during the experimental period. The incidence table is as follows: southern catfish with caudal fin congestion; bleeding, ulceration, skin blackening, moulting, and other symptoms are counted as the disease (Table 2):

### 3.2. Data Analysis of Transcriptome

From Illumina sequencing, a total of 79.37 Gb clean data were quality-filtered from 89.46 Gb of raw data (Table 3). The average length of merged transcripts was 1918 bp, and the N50 length was 32598 bp.

### 3.3. Differentially Expressed Genes (DEGs) Regulated by Exposure of P. shigelloides and Lactobacillus

**Figure 2:**
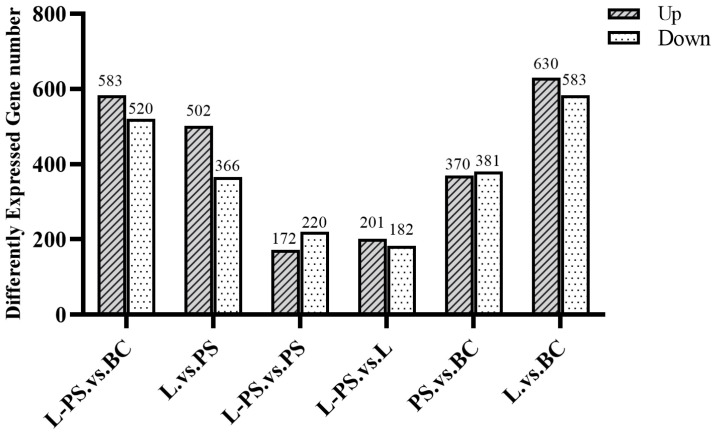
DEG statistics for all groups.

In total, 370 upregulated and 381 downregulated DEGs were detected in the PS group vs. the BC group, 630 upregulated and 583 downregulated DEGs were detected in the L group vs. the BC group, 583 upregulated and 520 downregulated DEGs were detected in the L-PS group vs. the BC group, and 172 upregulated and 220 downregulated DEGs were detected in the L-PS group vs. the PS group (Figure 2).

### 3.4. Gene Ontology Enrichment Analysis for DEGs

**Figure 3:**
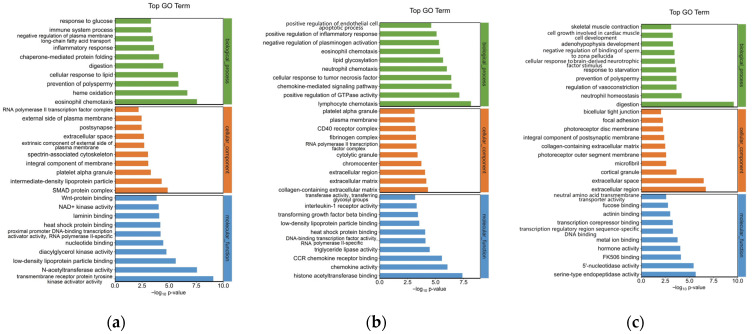
GO functional analysis of differentially expressed genes in spleen tissues of southern catfish. (**a**) Top 30 enriched GO terms of upregulated DEGs between PS and BC groups. (**b**) Top 30 enriched GO terms of downregulated DEGs between L-PS and PS groups. (**c**) Top 30 enriched GO terms of downregulated DEGs between L and BC groups.

To explore the mechanism of the southern catfish’s immune response to *P. shigelloides* at the genetic level, 751 DEGs were processed for GO enrichment analysis between the BC and PS groups. The top 30 enriched GO terms of upregulated genes are presented in Figure 3a. The immune-related GO terms were significantly (*p* < 0.05) enriched in the spleens of southern catfish infected with *P. shigelloides*, such as the inflammatory response, immune system process, and platelet alpha granules.

To explore the mechanism of *Lactobacillus* supplementation at the genetic level, 392 DEGs were processed for GO enrichment analysis between the L-PS and PS groups. Inflammation-related GO terms were significantly (*p* < 0.05) enriched, such as lymphocyte chemotaxis, positive regulation of inflammatory response, and interleukin-1 receptor activity. The top 30 enriched GO terms of downregulated DEGs are presented in Figure 3b. In the L group vs. the BC group, significantly upregulated genes (*p* < 0.05) were enriched in neutrophil homeostasis, regulation of vasoconstriction, and so on (Figure 3c).

### 3.5. KEGG Analysis for DEGs

**Figure 4:**
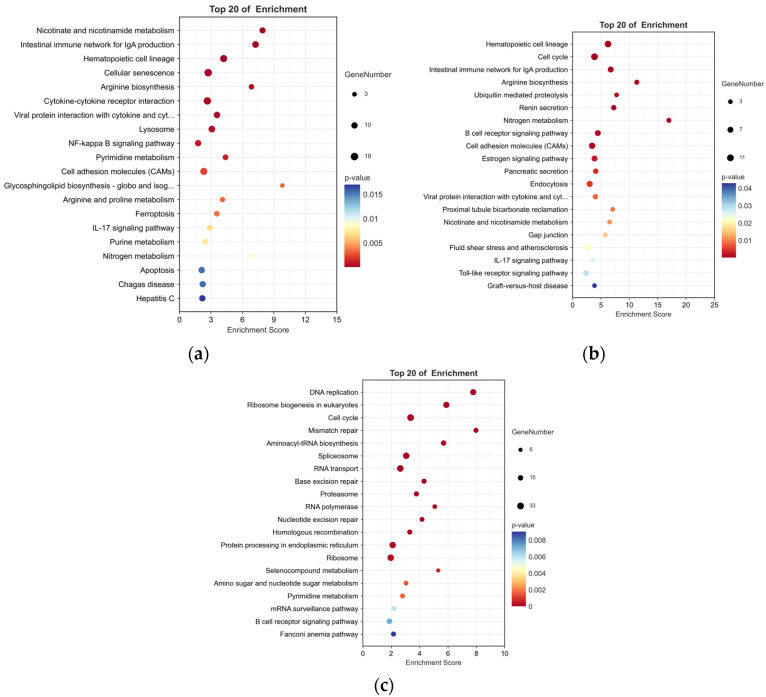
KEGG highly significant enrichment pathways of DEGs between two pairwise comparisons. (**a**) Top 20 pathways enriched in upregulated DEGs between PS and BC groups. (**b**) Top 20 pathways enriched in downregulated DEGs between L-PS and PS groups. (**c**) Top 20 pathways enriched in downregulated DEGs between L and BC groups.

To examine the pathways affected by *P. shigelloides*, 751 DEGs were processed for KEGG pathway enrichment analysis between the PS and BC groups. Some immune-related pathways, such as the intestinal immune network for IgA production, IL-17 signalling pathway, and so on, were significantly (*p* < 0.05) enriched in upregulated DEGs (Figure 4a).

To explore the mechanism of *Lactobacillus* supplementation at the genetic level, 392 DEGs were processed for KEGG enrichment analysis between the L-PS and PS groups. In the L-PS vs. the PS group, significantly downregulated genes (*p* < 0.05) were also enriched in the intestinal immune network for IgA production and the IL-17 signalling pathway (Figure 4b). In the L group vs. the BC group, significantly downregulated genes (*p* < 0.05) were enriched in the B-cell receptor signalling pathway, which was the same as the results in the L-PS group vs. the PS group (Figure 4c).

### 3.6. Analysis of Immune-Related Pathways

#### 3.6.1. IL-17 Signalling Pathway

**Figure 5:**
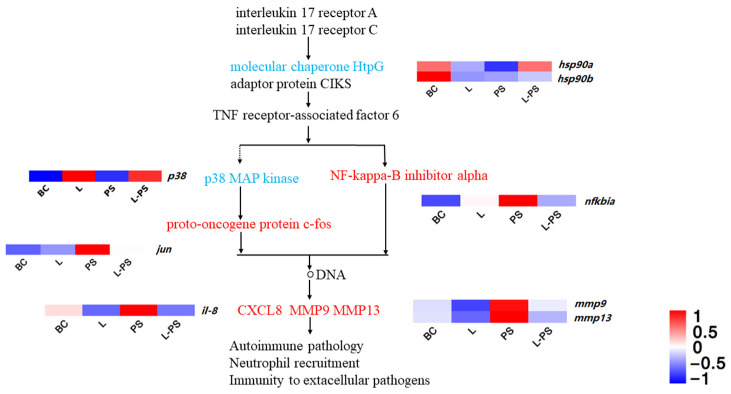
IL-17 signalling pathway.

In the IL-17 signalling pathway, our results showed that the expression of proinflammatory factors within the range was increased, and the trend between groups, such as *mmp9*, *il-8*, *jun*, and *mmp13*, was significantly upregulated (*p* < 0.05) in the PS group vs. the BC group, and *hsp90* was significantly downregulated (*p* < 0.05) in the PS group vs. the BC group. In the L-PS group vs. the PS group, *mmp9* and *mmp13* were significantly downregulated (*p* < 0.05), and *hsp90* was significantly upregulated (*p* < 0.05), while *il-8* expression was downregulated (*p* < 0.05). In the L group vs. the BC group, the expression of *mmp9* and *mmp13* was significantly downregulated (*p* < 0.05) (Figure 5).

#### 3.6.2. TNF Signalling Pathway

**Figure 6:**
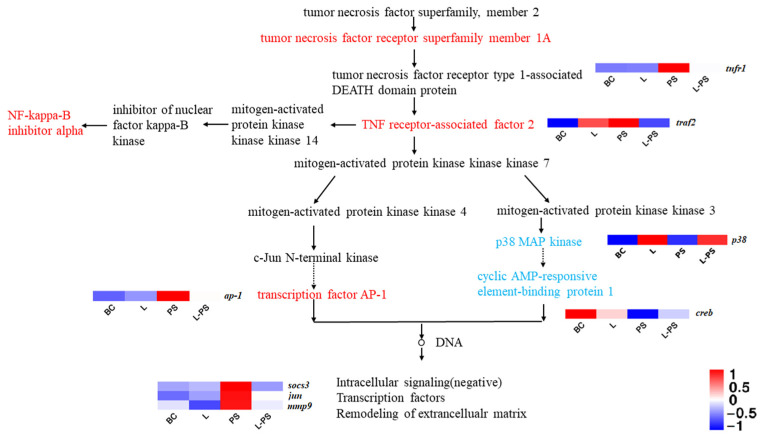
TNF signalling pathway.

In the TNF signalling pathway, the results showed that the expression of *socs3*, *tnfr1*, *traf2*, and *ap-1* was significantly upregulated (*p* < 0.05) in the PS group vs. the BC group; however, the expression of *p38* and *creb* was significantly downregulated (*p* < 0.05). In the L-PS group vs. the PS group, the expression of *socs3* and *mmp9* was significantly downregulated (*p* < 0.05), while the expression of *tnfr1*, *traf2*, *ap-1*, and *jun* was downregulated (Figure 6). In the L group vs. the BC group, *p38* expression was significantly upregulated (*p* < 0.05).

#### 3.6.3. Toll-like Receptor Signalling Pathway

**Figure 7:**
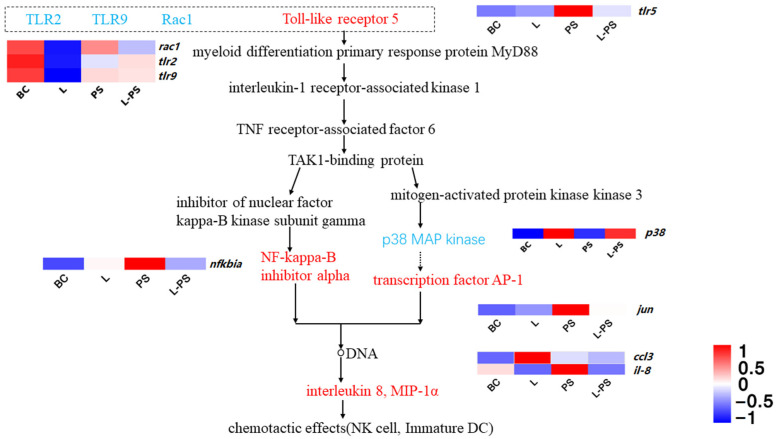
Toll-like receptor signalling pathway.

The results showed that several proinflammatory factors, such as *tlr5*, *nfkbia*, and *ccl3*, were significantly (*p* < 0.05) upregulated in the PS group vs. the BC group. In the L-PS group vs. the PS group, the expression of *tlr5*, *jun*, and *nfkbia* was downregulated; however, *p38* expression was significantly (*p* < 0.05) upregulated. In the L group vs. the BC group, *ccl3* expression was significantly (*p* < 0.05) upregulated, while the expression of *rac1*, *tlr2*, and *tlr9* was significantly (*p* < 0.05) downregulated (Figure 7).

#### 3.6.4. Intestinal Immune Network for IgA Production

**Figure 8:**
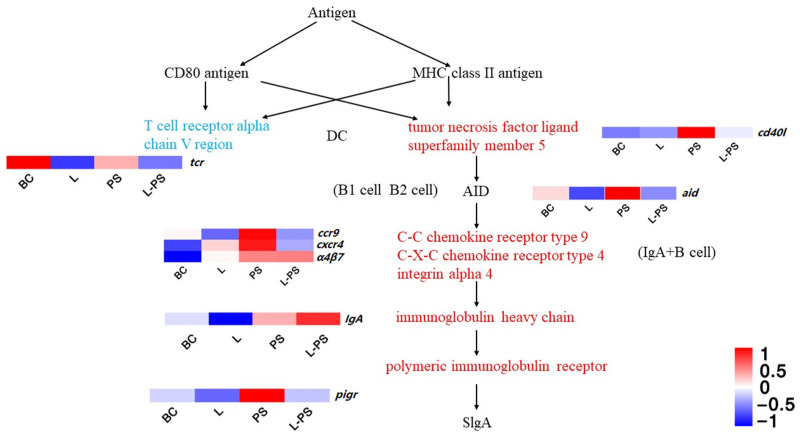
Intestinal immune network for IgA production.

In this signalling pathway, the expression of *cxcr4*, *pigr*, *ccr9*, *α4β7*, and *cd40l* was significantly upregulated (*p* < 0.05) in the PS group vs. the BC group. In the L-PS group vs. the PS group, the expression of *cxcr4*, *pigr*, *ccr9*, *tcr*, and *aid* was significantly downregulated (*p* < 0.05) (Figure 8). In the L-PS group vs. the BC group, the expression of *ccr9* and *cxcr4* had no significant difference.

### 3.7. Validation of DEGs by qRT-PCR

**Figure 9:**
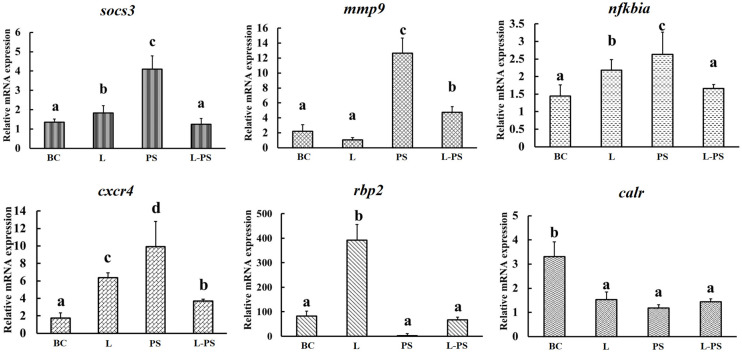
Validation of DEGs by qRT-PCR. BC: blank control group; L: Lactobacillus group; PS: *P. shigelloides* group; L-PS: *Lactobacillus* + *P. shigelloides* group; different characters (a, b, c, d) show significant difference (*p* < 0.05).

**Figure 10:**
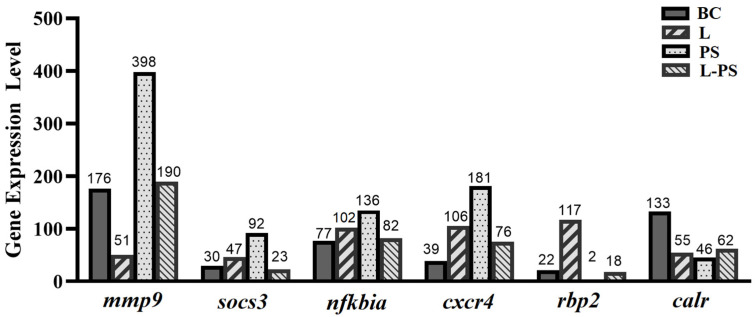
Expression levels of six differentially expressed genes obtained through RNA-Seq.

To validate the expression profiles of genes identified through RNA-Seq, we analysed the relative mRNA levels of the following six differentially expressed genes (*mmp9*, *socs3*, *cxcr4*, *nfkbia*, *rbp2*, and *calr*) by qRT-PCR (Figure 9). Our results showed that the qRT-PCR results and the results obtained through RNA-Seq were consistent (Figure 10).

### 3.8. Validation of DEGs by ELISA

**Figure 11:**
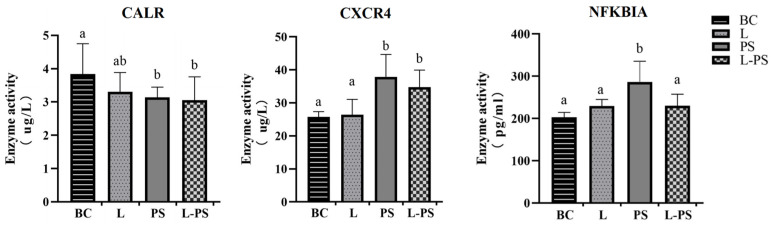
Protein levels of CXCR4, NFKBIA, and CALR in four groups. Different characters (a, b, c, d) show significant difference (*p* < 0.05).

To further confirm the changes in the spleens, we used ELISA to detect the contents of CXCR4, CALR, and NFKBIA in the spleen tissue homogenate. The ELISA results showed that the concentrations of CXCR4 and NFKBIA proteins were significantly (*p* < 0.05) upregulated in the PS group compared with the other groups. The concentrations of the CALR protein were significantly (*p* < 0.05) downregulated between the PS and BC groups. The trends of the three proteins were consistent with the RNA expression and transcriptome data (Figure 11).

## 4. Discussion

The spleen, an important organ in a fish’s immune system, is considered a primordial secondary lymphoid organ where an adaptive immune response is produced [45]. Bacterial diseases are extremely harmful to animals in large-scale aquaculture facilities. Once an infection occurs, these diseases spread rapidly. At present, the most common treatment for bacterial diseases is the use of antibiotics. However, the long-term use of antibiotics will not only pollute the water body but also make the intestinal flora of the fish more resistant, making the treatment of fish diseases more difficult in the future. Therefore, it is of great significance to explore effective and green control methods.

### 4.1. Probiotic Complex Had Inhibitory Effects on Inflammatory Response Caused by P. shigelloides in Southern Catfish

#### 4.1.1. IL-17 Signalling Pathway

IL-17 is a proinflammatory cytokine specifically secreted by helper T cells that can induce the secretion of early immune mediators [46,47]. Its family members, IL-17A and IL-17F, activate the downstream IK-Ba through IL-17R-Act1-TRAF6 and indirectly act on MAPKs to activate AP-1. A sustained inflammatory response can increase the expression of NF-κB, activate downstream chemokines and other effector genes of the NF-κB signalling pathway to increase the expression, and ultimately act on biological processes, such as neutrophil recruitment [48,49]. A high expression of matrix metalloproteinases can also indicate that the body is in a state of inflammation. Matrix metalloproteinase-9 (*mmp9*) is an important member of the MMP family, also known as gelatinase B [50]. A high expression of *mmp9* under pathological conditions reshapes the extracellular matrix through excessive degradation, exposes relevant active sites, destroys the physiological barrier, and aggravates the inflammatory response [51]. Studies have confirmed that the upregulation of *mmp9* is closely related to the body’s inflammatory response process [52,53,54]. In this study, the *mmp9* gene was significantly (*p* < 0.05) expressed in the PS group, which proved that *mmp9* may be involved in the pathogenesis of the inflammatory response caused by *P. shigelloides*. Combined with the comprehensive analysis of other groups, the expression of *mmp9* in the L group and the L-PS group was significantly (*p* < 0.05) reduced, indicating that the probiotic complex had an inhibitory effect on the expression of this gene; therefore, it could inhibit the inflammatory response to a certain extent.

Neutrophils are the first line of immune defence and are phagocytes that play a crucial role in the early immune response of the host by clearing pathogens [55,56]. *il-8* is a member of the α subfamily of chemokines, which acts on neutrophil chemotaxis, promotes T-cell chemotaxis and migration, and strengthens the immune response [57]. In our study, the reason for the significant expression of *il-8* in the PS group may be due to the inflammatory response of the southern catfish infected with *P. shigelloides*, which activates macrophages and promotes the chemotaxis of neutrophils to the site of inflammation. Compared with the BC group, *il-8* expression in the L group was significantly (*p* < 0.05) reduced, indicating that the expression of proinflammatory factors could be inhibited after treatment with the probiotic complex, thereby inhibiting the inflammatory response. Comparing the L-PS group and the PS group, we found that the expression of *il-8* was significantly (*p* < 0.05) reduced, indicating that the feeding of probiotics may enhance the resistance of southern catfish to pathogenic invasion and that the probiotic complex can effectively inhibit the inflammatory response caused by the invasion of *P. shigelloides*.

#### 4.1.2. TNF Signalling Pathway

The TNF (tumour necrosis factor), also known as TNFα, is a cytokine that can directly kill tumour cells and has no obvious cytotoxicity to normal cells. It is a cell signalling protein involved in systemic inflammation and a cytokine that constitutes the acute phase response [58]. The expression of the downstream gene *socs3* in this pathway is also regulated to varying degrees. SOCS3 is one of the important members of the SOCS family proteins. *socs3* has been certified to play a key role in immune response in aquatic animals, such as *Cynoglossus semilaevis* [59], *Paralichthys olivaceus* [60], and *Ictalurus punctatus* [61]. In our study, *socs3* gene expression was significantly upregulated (*p* < 0.05) in the PS vs. BC group, indicating that *P. shigelloides* could induce the expression of *socs3*. Studies have also shown that *socs3* expression can be induced by the lipopolysaccharides of Gram-negative bacteria [62]. In the L-PS vs. PS group, the expression of *socs3* was significantly downregulated. We speculated that feeding of the probiotic complex had a protective effect on southern catfish.

The TNF is involved in the innate immune response through two receptors, TNFR1 and TNFR2. In this study, the significant expression of *tnfr1* in the PS group showed that infection with *P. shigelloides* stimulated an immune response and activated the TNFR1 signalling pathway (TNFR1-TRADD-TRAF), which inhibits apoptosis and promotes inflammation. Because TNFα is associated with an acute inflammatory response, it may be the reason for the lack of a significant difference in *tnfr1* expression between the L group and the BC group. Compared with the PS group, the expression of *tnfr1* in the L-PS group was significantly (*p* < 0.05) decreased, indicating that long-term feeding of probiotics may effectively respond to the *TNFα*-mediated inflammatory pathway caused by the invasion of *P. shigelloides* and inhibit the inflammatory response to a certain extent.

#### 4.1.3. Toll-like Receptor Signalling Pathway

The cytoplasmic TIR domain of TLR recruits signal adaptors MyD88, TIRAP, TRAM, and/or TRIF and activates various kinases (IRAK4, IRAK1, IRAK2, TBK1, and IKKε) and ubiquitin ligases (TRAF6 and pellino 1), thereby activating nuclear factor NF-κB and inducing the expression of inflammatory factors. *nfkbia* is the gene encoding the NF-κB complex inhibitory protein IκB [63]. There are related studies involving this gene in aquatic animals. *nfkbia* gene expression in zebrafish juveniles infected with *Vibrio parahaemolyticus* [64], *Lampetra japonica* stimulated by dsRNA virus mimetic [65], and *Channa argus* after infection with *Americide nocardia* [66] were significantly (*p* < 0.05) upregulated. In this study, *nfkbia* expression in the PS group was significantly (*p* < 0.05) upregulated compared with the BC group, consistent with the findings above, indicating that *nfkbia* may be involved in the early generation of the innate immune response to *P. shigelloides* infection in the southern catfish.

In the TLR family, TLR5, the receptor of flagellin, which is related to the pathogenicity of bacteria, has a defensive effect against the invasion of flagella [67]. In our study, the expression of *tlr5* in the PS group was significantly (*p* < 0.05) upregulated, which may be due to the surface structure of bacteria inducing the body to produce a large number of TLR5 receptor proteins to resist bacterial invasion, indicating that *P. shigelloides* infection can cause TLR5-mediated innate immunity in the southern catfish. The expression of *tlr5* in the L group was not significantly (*p* < 0.05) different from that in the BC group, which may be because probiotics have little effect on *tlr5* regulation without pathogenic invasion. Compared with the PS group, the expression of *tlr5* in the L-PS group was significantly (*p* < 0.05) reduced, and we hypothesised that this may have been due to the enhanced immunity and disease resistance of the southern catfish due to the probiotics. In addition, it is possible that the number of probiotics in the body is large and that the produced metabolites inhibit the growth of *P. shigelloides*. We performed a series of in vitro bacteriostatic experiments and showed that different probiotic metabolite concentrations can inhibit the growth of *P. shigelloides* to varying degrees.

#### 4.1.4. Intestinal Immune Network for IgA Production

CXCR4 (chemokine receptor 4) is an inflammatory chemokine that acts by binding to its ligand SDF-1 (stromal cell-derived factor-1). The possible mechanism of the SDF-1/CXCR4 axis-mediated inflammatory response is chemotaxis of neutrophils and other inflammatory cells to the site of inflammation [68]. In our study, the expression of *cxcr4* in the PS group was significantly (*p* < 0.05) increased, indicating that the invasion of *P. shigelloides* caused the southern catfish to be in an inflammatory state. In the L-PS vs. the PS group, the *cxcr4* gene expression was significantly (*p* < 0.05) downregulated. Our results showed that the degree of inflammatory response in the L-PS group was lighter compared with the PS group. In addition, we detected the concentration of CXCR4 protein by ELISA, and the change trend was consistent with the change trend of *cxcr4* expression.

pIgR (poly-Ig receptor) is involved in the innate immunity of the organism mucosa. pIgR mediates IgA transportation to neutralise extracellular pathogens in mammals [69,70]. To some extent, the expression level of pIgR in mucosal epithelium represents the expression level of IgA antibody in extra mucosal secretions [71]. In teleost fish, pIgR can transport IgM and IgT to mucosal secretions and exert its mucosal immune function [72,73]. Studies have also shown that IgT in fish can mediate the intestinal lumen through pIgR, which is considered a specific immunoglobulin involved in intestinal mucosal immunity, and its function is similar to mammalian IgA [74]. Studies have shown that the expression of *pigr* in the spleen of *Scophthalmus maxima* is significantly (*p* < 0.05) upregulated after infection by *Vibrio anguillarum* [75]. The expression of *pigr* in the spleen of zebrafish was also significantly (*p* < 0.05) upregulated after infection by *Streptococcus iniae* [76]. In our study, the expression of *pigr* was significantly (*p* < 0.05) upregulated in the PS group compared with the BC group, indicating that the invasion of *P. shigelloides* can lead to the activation of the intestinal immune network for IgA production, upregulating the downstream key gene *pigr* to promote inflammatory response. The expression of some transcripts of the *pigr* gene in the L vs. the BC group was downregulated, and *pigr* gene expression was significantly (*p* < 0.05) downregulated in the L-PS vs. the PS group. The above results show that the probiotic complex could inhibit the expression of *pigr* to relieve inflammation.

### 4.2. Other Related Key Genes

Calreticulin (CALR) protein can maintain intracellular calcium homeostasis and assist in protein folding, and plays a significant role in immune regulation, cell phagocytosis, cell proliferation, and apoptosis [77]. In our study, the expression of *calr* in the L, PS, and L-PS groups was significantly (*p* < 0.05) lower than the BC group; however, CALR protein concentration in the PS and L-PS groups was lower than that in BC and L groups, probably because the invasion of *P. shigelloides* could inhibit the function of CALR. RBP2 plays a key role in maintaining the normal DC response, which has functions in phagocytosis, processing, and presenting antigens [78]. In our study, we hypothesised that the downregulation of *rbp2* in the PS group may be related to the mechanism of *P. shigelloides* invasion to evade immunity. MHCI cytotoxic T lymphocytes (CD8+) play a key role in the recognition of antigen peptide–MHC complexes [79]. In this study, *mhc I* expression was significantly (*p* < 0.05) downregulated in the PS group. We speculated that it was because *P. shigelloides* inhibited *mhc I* expression to evade the body’s immune system; however, this conjecture needs to be further verified. Arachidonate 5-lipoxygenase (Alox5) is a membrane-bound protein mainly expressed in polymorphonuclear leukocytes, peripheral blood monocytes, macrophages, and mast cells. It is highly expressed in many diseases [80,81,82]. Inhibition of *alox5* expression attenuates lipopolysaccharide (LPS)-induced inflammation [83]. In our study, the expression of *alox5* in the L and L-PS groups was significantly (*p* < 0.05) downregulated, which also showed that the probiotic complex could inhibit the expression of *alox5* and block the production of inflammatory mediators (Supplementary Figure S1 [84,85,86]).

## 5. Conclusions

In conclusion, in our experiment, we found that *P. shigelloides* can cause hyperaemia and the festering of fins, while southern catfish fed a probiotic complex were free of related inflammation after the infection of *P. shigelloides*. Moreover, significantly downregulated genes (*p* < 0.05) were enriched in inflammation-related GO terms (lymphocyte chemotaxis and positive regulation of inflammatory response) and immune-related pathways (intestinal immune network for IgA production and IL-17 signalling pathway) in the L-PS vs. the PS group. Hence, probiotics could inhibit the inflammatory response caused by *P. shigelloides* to some extent. Therefore, probiotics are promising alternatives to antibiotics to prevent bacterial diseases in the aquaculture industry.

## Figures and Tables

**Table 1:** Specific primers list.

Gene Symbol	Forward Primer (5′->3′)	Reverse Primer (5′->3′)
18s rRNA	GTGTCCGGTCCCTTTCAG	CAGGTATTCAGGCGAGTT
β-actin	CAGACGCTACTTCGAGTTT	CTCATCCATGGCGATGAAT
*mmp9*	TGAATTGTCCCAACTACCGAA	ACATCTGGGATTTGCATTTAAG
*socs3*	CAGTACCCAAATGACTAGGAAC	CCCTGAAAGGCATAATGCT
*nfkbia*	ACACCAAACTACAACGGTC	CGTTACACTGCTCCTGTTC
*cxcr4*	CCGTTCCTGATCTGGTCTT	TTGGCTTCGTGAGGGTAG
*rbp2*	GTGCGCAATGAGAACTTTGA	GTTTGTGTGAGGTGTGCT
*calr*	TCAACTCGGTTTGAGGACTT	ATCCTCCTCCACAGTCTATG

**Table 2:** Incidence of southern catfish in each group.

Group	Number	Patients	Prevalence
BC	20	0	0%
L	20	0	0%
PS	20	17	85%
L-PS	20	3	15%

Note: BC: blank control group; L: *Lactobacillus* group; PS: *P. shigelloides* group; L-PS: *Lactobacillus*-*P. shigelloides* group.

**Table 3:** Summary of RNA-sequencing results and quality data output.

Sample	Raw Reads	Raw Bases	Clean Reads	Clean Bases	Valid Bases	Q30	GC
BC1	48.21M	7.28G	47.13M	6.53G	89.73%	93.87%	49.29%
BC2	49.20M	7.43G	48.15M	6.69G	90.07%	93.99%	49.52%
BC3	47.87M	7.23G	46.83M	6.51G	90.09%	93.88%	49.21%
L1	48.63M	7.34G	47.53M	6.60G	89.91%	93.81%	49.44%
L2	49.67M	7.50G	48.63M	6.71G	89.44%	94.07%	49.81%
L3	50.32M	7.60G	49.25M	6.86G	90.24%	93.92%	49.12%
L_PS1	49.03M	7.40G	47.75M	6.49G	87.59%	93.39%	49.02%
L_PS2	49.34M	7.45G	48.05M	6.53G	87.69%	93.36%	49.06%
L_PS3	49.59M	7.49G	48.26M	6.55G	87.42%	93.23%	49.30%
PS1	51.08M	7.66G	49.66M	6.70G	87.46%	93.15%	49.43%
PS2	48.48M	7.32G	47.13M	6.43G	87.82%	93.64%	49.41%
PS3	51.37M	7.76G	49.95M	6.77G	87.32%	93.36%	49.15%

Note: BC: blank control group; L: *Lactobacillus* group; PS: *P. shigelloides* group; L-PS: *Lactobacillus*-*P. shigelloides* group.

## Data Availability

The raw sequence data reported in this paper have been deposited in the Genome Sequence Archive (Genomics, Proteomics & Bioinformatics 2021) in the National Genomics Data Center (Nucleic Acids Res 2022), China National Center for Bioinformation/Beijing Institute of Genomics, and the Chinese Academy of Sciences (GSA: CRA008891) and are publicly accessible at https://ngdc.cncb.ac.cn/gsa, accessed on 15 November 2022.

## Supplementary Materials

The following supporting information can be downloaded at: https://www.mdpi.com/article/10.3390/ani13030449/s1, Figure S1: Arachidonic acid metabolism.
