# Supplementary material for: Transcriptome Analysis Reveals Effect of Dietary Probiotics on Immune Response Mechanism in Southern Catfish (Silurus meridionalis) in Response to Plesiomonas shigelloides
Source: Animals (Basel). 2023 Jan 28;13(3):449. doi: 10.3390/ani13030449 (PMC9913393; doi:10.3390/ani13030449)
Supplement: Supplementary file 1 [file animals-13-00449-s001.zip › animals-2083742-supplementary.pdf]

Supplementary materials:

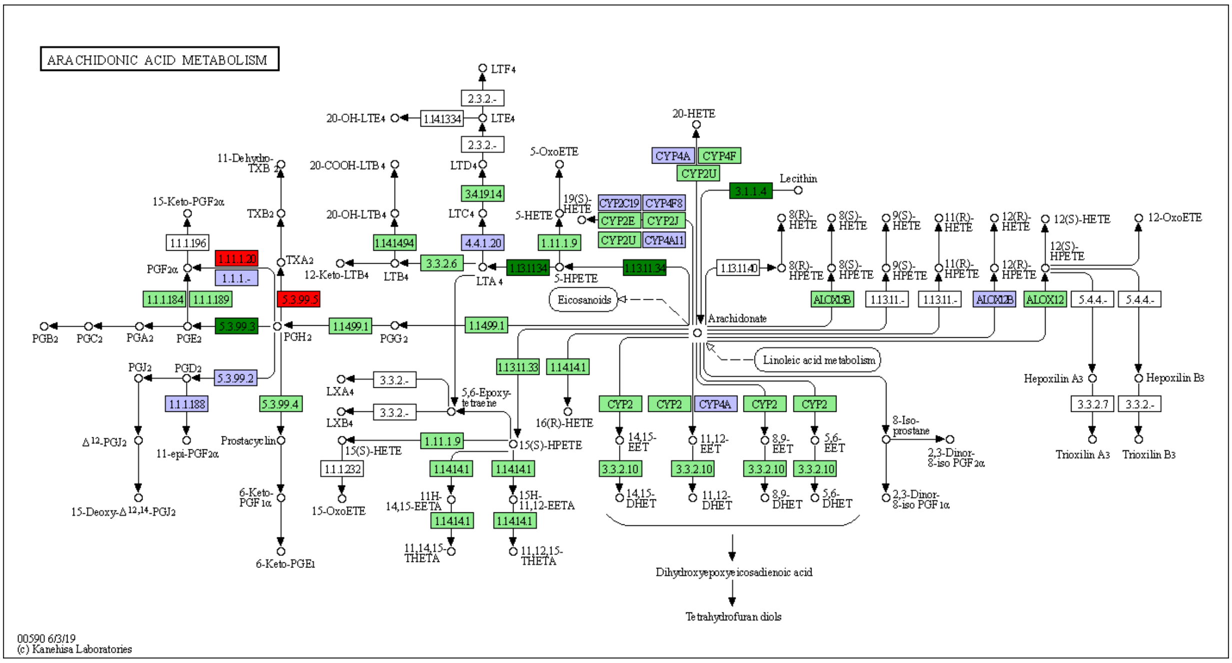

(**a**)

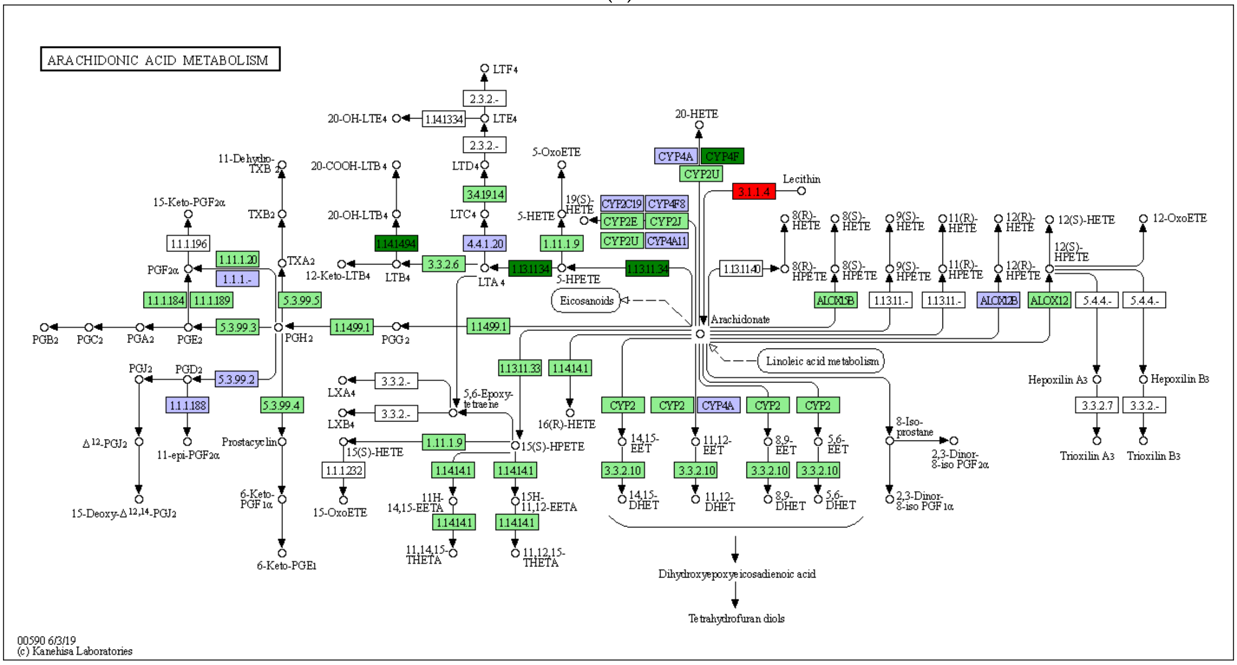

(**b**)

Figure S1. Arachidonic acid metabolism (Reproduced with permission from [Kanehisa Laboratories], [KEGG: kyoto encyclopedia of genes and genomes]; published by [Kanehisa and S Goto], [2000]). (**a**) The map of Arachidonic acid metabolism in L vs. BC group; 1.13.11.34: Arachidonate 5-lipoxygenase (Alox5) [84–86]. (**b**) The map of Arachidonic acid metabolism in L-PS vs. PS group; 1.13.11.34: Arachidonate 5-lipoxygenase (Alox5) [84–86].

References

84. Kanehisa, M.; Goto, S. Kegg: Kyoto encyclopedia of genes and genomes. *Nucleic Acids Res*. **2000**, *28*, 27–30.
85. Kanehisa, M. Toward understanding the origin and evolution of cellular organisms. *Protein Sci.: a publication of the Protein Society* **2019**, *28*, 1947–1951.

86. Kanehisa, M.; Furumichi, M.; Sato, Y.; Ishiguro-Watanabe, M.; Tanabe, M. KEGG: Integrating viruses and cellular organisms. *Nucleic Acids Res.* **2021**, *49*, D545–D551.
